# 1,3-Bis(2-methyl­prop-2-eno­yl)-1*H*-benz­imidazol-2(3*H*)-one

**DOI:** 10.1107/S1600536813011380

**Published:** 2013-05-04

**Authors:** N. Haridharan, V. Ramkumar

**Affiliations:** a Department of Chemistry, University College of Engineering Panruti (A Constituent College of Anna University), Panruti 607 106, Tamilnadu, India; b Department of Chemistry, IIT Madras, Chennai, TamilNadu, India

## Abstract

The mol­ecules of the title compound, C_15_H_14_N_2_O_3_, possesses crystallographically imposed twofold rotational symmetry, so the asymmetric unit contains one half-mol­ecule. The fused-ring system deviates significantly from planarity; the planes of the five- and six-membered rings are twisted with respect to each other by 3.0 (1)°. In the crystal, weak C—H⋯O hydrogen bonds link mol­ecules related by translation in [010] into chains.

## Related literature

For applications of substituted benzimidazoles, see: Gravatt *et al.* (1994 ▶); Srikanth *et al.* (2011 ▶). For the crystal structures of related compounds, see: Ouzidan *et al.* (2011 ▶); Kandri Rodi *et al.* (2011 ▶).
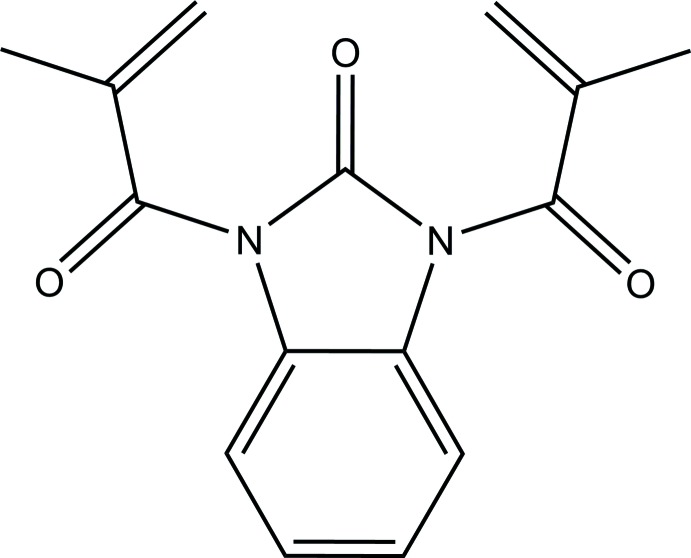


## Experimental

### 

#### Crystal data

C_15_H_14_N_2_O_3_
*M* *_r_* = 270.28
Monoclinic, 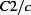

*a* = 16.6359 (9) Å
*b* = 8.8629 (5) Å
*c* = 9.6221 (4) Å
β = 102.775 (2)°
*V* = 1383.59 (12) Å^3^
*Z* = 4
Mo *K*α radiation
μ = 0.09 mm^−1^
*T* = 298 K
0.22 × 0.18 × 0.10 mm

#### Data collection

Bruker APEXII CCD area-detector diffractometer
Absorption correction: multi-scan (*SADABS*; Bruker, 2004 ▶) *T*_min_ = 0.980, *T*_max_ = 0.991
4086 measured reflections
1165 independent reflections
1042 reflections with *I* > 2σ(*I*)
*R*_int_ = 0.014

#### Refinement

*R*[*F* ^2^ > 2σ(*F* ^2^)] = 0.057
*wR*(*F* ^2^) = 0.208
*S* = 1.16
1165 reflections
93 parameters
2 restraints
H-atom parameters constrained
Δρ_max_ = 0.41 e Å^−3^
Δρ_min_ = −0.36 e Å^−3^

### 

Data collection: *APEX2* (Bruker, 2004 ▶); cell refinement: *SAINT-Plus* (Bruker, 2004 ▶); data reduction: *SAINT-Plus*; program(s) used to solve structure: *SHELXS97* (Sheldrick, 2008 ▶); program(s) used to refine structure: *SHELXL97* (Sheldrick, 2008 ▶); molecular graphics: *ORTEP-3 for Windows* (Farrugia, 2012 ▶); software used to prepare material for publication: *SHELXL97*.

## Supplementary Material

Crystal structure: contains datablock(s) global, I. DOI: 10.1107/S1600536813011380/cv5390sup1.cif

Structure factors: contains datablock(s) I. DOI: 10.1107/S1600536813011380/cv5390Isup2.hkl

Supplementary material file. DOI: 10.1107/S1600536813011380/cv5390Isup3.cml

Additional supplementary materials:  crystallographic information; 3D view; checkCIF report

## Figures and Tables

**Table 1:** Hydrogen-bond geometry (Å, °)

*D*—H⋯*A*	*D*—H	H⋯*A*	*D*⋯*A*	*D*—H⋯*A*
C1—H1⋯O1^i^	0.93	2.57	3.193 (3)	124

Symmetry code: (i) 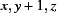.

**Fig. 1.**
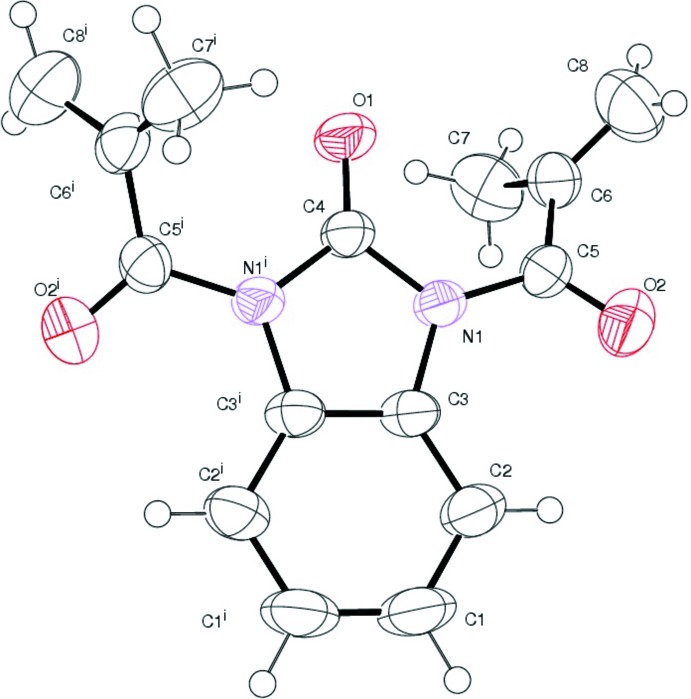
View of (I) showing the atomic numbering and 50% probability displacement ellipsoids [symmetry code: -x, y, 1/2 - z].

## supplementary crystallographic information

### Comment

Substituted benzimidazole derivatives have found wide range of therapeutic and pharmacological applications (Gravatt *et al.*, 1994; Srikanth *et al.*, 2011). Herewith we present the title compound, (I), which is a new derivative of benzimidazole.

In (I) (Fig. 1), all bond lengths and angles are normal and comparable with those reported for related compounds (Ouzidan *et al.*, 2011; Kandri Rodi *et al.*, 2011). The molecules in (I) possess a crystallographically imposed twofold rotational symmetry, with half of the molecule in the assymetric unit. The five- and six-membered rings are twisted with a dihedral angle of 3.0 (1)°.

In the crystal, weak intermolecular C—H···O hydrogen bonds (Table 1) link the molecules related by translation in [010] into chains.

### Experimental

2-Hydroxy benzimidazole (1 g, 0.007 moles) and THF (100 ml) were placed in a 3-neck round bottomed flask. Methacrylic anhydride (2.29 g, 0.014 moles) was added slowly, using a syringe, with stirring. The mixture was allowed to cool to 0 °C using ice-salt mixture with stirring. The mixture was treated with sodium hydroxide (0.56 g, 0.014 moles) drop by drop and the amide formation reaction was allowed to stir for 6 h at RT. The resulting crude product was dissolved in ethyl acetate, washed with bicarbonate solution and then with water thrice followed by brine solution and dried over anhydrous sodium sulfate. The resulting solvent was removed by rotary evaporation. The product was purified by column chromatography technique using 12% ethyl acetate in hexane as the eluent to obtain a product as a bright white solid. Recrystallization of the compound from acetone gave X-ray diffraction quality crystals of (I).

### Refinement

All hydrogen atoms were fixed geometrically and allowed to ride on the parent carbon atoms with aromatic C—H = 0.93 Å, methylene C—H = 0.97 Å and methyl C—H = 0.96 Å. The displacement parameters were set as *U*_iso_(H) = 1.2–1.5 *U*_eq_(C).

### Crystal data

**Table:**

C_15_H_14_N_2_O_3_	*F*(000) = 568
*M**_r_* = 270.28	*D*_x_ = 1.298 Mg m^−^^3^
Monoclinic, *C*2/*c*	Mo *K*α radiation, λ = 0.71073 Å
Hall symbol: -C 2yc	Cell parameters from 3023 reflections
*a* = 16.6359 (9) Å	θ = 2.6–28.4°
*b* = 8.8629 (5) Å	µ = 0.09 mm^−^^1^
*c* = 9.6221 (4) Å	*T* = 298 K
β = 102.775 (2)°	Prism, colourless
*V* = 1383.59 (12) Å^3^	0.22 × 0.18 × 0.10 mm
*Z* = 4	

### Data collection

**Table:**

Bruker APEXII CCD area-detector diffractometer	1165 independent reflections
Radiation source: fine-focus sealed tube	1042 reflections with *I* > 2σ(*I*)
Graphite monochromator	*R*_int_ = 0.014
phi and ω scans	θ_max_ = 25.0°, θ_min_ = 3.2°
Absorption correction: multi-scan (*SADABS*; Bruker, 2004)	*h* = −16→19
*T*_min_ = 0.980, *T*_max_ = 0.991	*k* = −10→10
4086 measured reflections	*l* = −11→7

### Refinement

**Table:**

Refinement on *F*^2^	Primary atom site location: structure-invariant direct methods
Least-squares matrix: full	Secondary atom site location: difference Fourier map
*R*[*F*^2^ > 2σ(*F*^2^)] = 0.057	Hydrogen site location: inferred from neighbouring sites
*wR*(*F*^2^) = 0.208	H-atom parameters constrained
*S* = 1.16	*w* = 1/[σ^2^(*F*_o_^2^) + (0.1442*P*)^2^ + 0.5014*P*] where *P* = (*F*_o_^2^ + 2*F*_c_^2^)/3
1165 reflections	(Δ/σ)_max_ < 0.001
93 parameters	Δρ_max_ = 0.41 e Å^−^^3^
2 restraints	Δρ_min_ = −0.36 e Å^−^^3^

### Special details

**Table:**

Geometry. All e.s.d.'s (except the e.s.d. in the dihedral angle between two l.s. planes) are estimated using the full covariance matrix. The cell e.s.d.'s are taken into account individually in the estimation of e.s.d.'s in distances, angles and torsion angles; correlations between e.s.d.'s in cell parameters are only used when they are defined by crystal symmetry. An approximate (isotropic) treatment of cell e.s.d.'s is used for estimating e.s.d.'s involving l.s. planes.
Refinement. Refinement of *F*^2^ against ALL reflections. The weighted *R*-factor *wR* and goodness of fit *S* are based on *F*^2^, conventional *R*-factors *R* are based on *F*, with *F* set to zero for negative *F*^2^. The threshold expression of *F*^2^ > σ(*F*^2^) is used only for calculating *R*-factors(gt) *etc*. and is not relevant to the choice of reflections for refinement. *R*-factors based on *F*^2^ are statistically about twice as large as those based on *F*, and *R*- factors based on ALL data will be even larger.

### Fractional atomic coordinates and isotropic or equivalent isotropic displacement parameters (Å^2^)

**Table:**

	*x*	*y*	*z*	*U*_iso_*/*U*_eq_	
C1	0.47013 (18)	0.8707 (3)	0.7898 (4)	0.0750 (10)	
H1	0.4508	0.9622	0.8167	0.090*	
C2	0.43851 (17)	0.7372 (3)	0.8301 (3)	0.0601 (8)	
H2	0.3981	0.7370	0.8832	0.072*	
C3	0.46936 (12)	0.6045 (2)	0.7882 (2)	0.0444 (6)	
C4	0.5000	0.3574 (3)	0.7500	0.0437 (8)	
C5	0.38571 (13)	0.4045 (2)	0.8743 (2)	0.0461 (7)	
C6	0.34437 (13)	0.2572 (2)	0.8323 (2)	0.0529 (7)	
N1	0.44820 (10)	0.45224 (19)	0.80638 (19)	0.0430 (6)	
O1	0.5000	0.2222 (2)	0.7500	0.0654 (8)	
O2	0.36526 (12)	0.4856 (2)	0.9606 (2)	0.0703 (7)	
C7	0.31257 (19)	0.2319 (4)	0.6791 (2)	0.0781 (10)	
H7A	0.3577	0.2117	0.6345	0.117*	
H7B	0.2836	0.3201	0.6371	0.117*	
H7C	0.2757	0.1472	0.6655	0.117*	
C8	0.3311 (2)	0.1649 (4)	0.9330 (3)	0.0889 (11)	
H8A	0.2999	0.0779	0.9084	0.107*	
H8B	0.3529	0.1870	1.0284	0.107*	

### Atomic displacement parameters (Å^2^)

**Table:**

	*U*^11^	*U*^22^	*U*^33^	*U*^12^	*U*^13^	*U*^23^
C1	0.0787 (19)	0.0326 (12)	0.117 (3)	0.0049 (10)	0.0282 (17)	−0.0064 (13)
C2	0.0603 (15)	0.0416 (13)	0.0812 (19)	0.0069 (10)	0.0217 (14)	−0.0045 (11)
C3	0.0411 (11)	0.0326 (12)	0.0578 (14)	0.0014 (7)	0.0071 (10)	0.0007 (8)
C4	0.0413 (15)	0.0329 (14)	0.0592 (19)	0.000	0.0162 (13)	0.000
C5	0.0396 (11)	0.0463 (13)	0.0546 (14)	0.0070 (8)	0.0152 (10)	0.0035 (9)
C6	0.0398 (12)	0.0520 (13)	0.0710 (17)	−0.0020 (9)	0.0208 (11)	0.0046 (10)
N1	0.0389 (9)	0.0329 (10)	0.0599 (12)	0.0014 (6)	0.0166 (8)	0.0003 (7)
O1	0.0647 (16)	0.0316 (13)	0.111 (2)	0.000	0.0435 (15)	0.000
O2	0.0726 (13)	0.0674 (12)	0.0816 (14)	0.0023 (9)	0.0399 (11)	−0.0090 (9)
C7	0.0629 (17)	0.077 (2)	0.089 (2)	−0.0211 (13)	0.0050 (16)	0.0023 (15)
C8	0.100 (2)	0.081 (2)	0.095 (2)	−0.0197 (18)	0.0398 (19)	0.0097 (17)

### Geometric parameters (Å, º)

**Table:**

C1—C1^i^	1.382 (6)	C5—O2	1.203 (3)
C1—C2	1.385 (4)	C5—N1	1.410 (3)
C1—H1	0.9300	C5—C6	1.489 (3)
C2—C3	1.378 (3)	C6—C8	1.3242 (19)
C2—H2	0.9300	C6—C7	1.4689 (19)
C3—C3^i^	1.383 (4)	C7—H7A	0.9600
C3—N1	1.415 (3)	C7—H7B	0.9600
C4—O1	1.198 (4)	C7—H7C	0.9600
C4—N1	1.397 (2)	C8—H8A	0.9300
C4—N1^i^	1.397 (2)	C8—H8B	0.9300
			
C1^i^—C1—C2	121.28 (17)	C8—C6—C7	124.0 (3)
C1^i^—C1—H1	119.4	C8—C6—C5	119.0 (2)
C2—C1—H1	119.4	C7—C6—C5	116.65 (19)
C3—C2—C1	117.3 (3)	C4—N1—C5	125.50 (19)
C3—C2—H2	121.4	C4—N1—C3	109.53 (17)
C1—C2—H2	121.4	C5—N1—C3	124.95 (17)
C2—C3—C3^i^	121.42 (15)	C6—C7—H7A	109.5
C2—C3—N1	131.2 (2)	C6—C7—H7B	109.5
C3^i^—C3—N1	107.37 (11)	H7A—C7—H7B	109.5
O1—C4—N1	127.01 (12)	C6—C7—H7C	109.5
O1—C4—N1^i^	127.01 (12)	H7A—C7—H7C	109.5
N1—C4—N1^i^	106.0 (2)	H7B—C7—H7C	109.5
O2—C5—N1	119.4 (2)	C6—C8—H8A	120.0
O2—C5—C6	121.8 (2)	C6—C8—H8B	120.0
N1—C5—C6	118.77 (18)	H8A—C8—H8B	120.0
			
C1^i^—C1—C2—C3	0.3 (6)	N1^i^—C4—N1—C3	−1.51 (11)
C1—C2—C3—C3^i^	1.4 (5)	O2—C5—N1—C4	153.7 (2)
C1—C2—C3—N1	−177.0 (2)	C6—C5—N1—C4	−28.9 (3)
O2—C5—C6—C8	−46.6 (4)	O2—C5—N1—C3	−24.6 (3)
N1—C5—C6—C8	136.0 (3)	C6—C5—N1—C3	152.8 (2)
O2—C5—C6—C7	126.7 (3)	C2—C3—N1—C4	−177.4 (2)
N1—C5—C6—C7	−50.7 (3)	C3^i^—C3—N1—C4	4.0 (3)
O1—C4—N1—C5	0.0 (2)	C2—C3—N1—C5	1.1 (4)
N1^i^—C4—N1—C5	180.0 (2)	C3^i^—C3—N1—C5	−177.4 (2)
O1—C4—N1—C3	178.48 (11)		

Symmetry code: (i) −*x*+1, *y*, −*z*+3/2.

### Hydrogen-bond geometry (Å, º)

**Table:**

*D*—H···*A*	*D*—H	H···*A*	*D*···*A*	*D*—H···*A*
C1—H1···O1^ii^	0.93	2.57	3.193 (3)	124

Symmetry code: (ii) *x*, *y*+1, *z*.
